# Odonto-Chirurgical Society of London England

**Published:** 1883-04

**Authors:** 


### ARTICLE III.

### *Odonto-Chirurgical Society, of London, England.*

The Ordinary Meeting of this Society was held 8th February, 1883, Dr. Smith, F. R. C. S. Ed., President, in the Chair.

The following Paper on "Alveolar Abscess, its Pathology and Treatment," was read by Mr. Watson, L. D. S. Edin. :—

Definition.—A collection of purulent matter contained in a sac attached to the radical extremity of the tooth, and formed or deposited in the alveoli or substance of the maxillary bones. (Varieties). Acute and Chronic.

Causes.—(*a*) Local. Mechanical injury from blows, resulting in loosening or fracture of tooth, injury produced by mechanical appliances in the treatment of Dental irregularities. Mechanical impact of the bite and caries, which is by far the most common cause of alveolar abscess.

(*b*) Constitutional.—Any of the Exanthemata, Pregnancy, Syphilis, Maleria, and certain medical substances, any of which, owing to the alteration or derivation of the nutrition from the dental organs, are liable to re-act on the periosteum and pulps of weak members of the denture, and so lead to alveolar abscess.

Pathology.—The irritation set up by a diseased root or tooth speedily affects the alveolar periosteum, produces hyperæmia and thickening of this membrane, raising the tooth in its socket and making it painful on pressure, then follows plastic exudation around the apex of root or roots, which through time becomes organised into a dense fibrous sac of connective tissue, the bundles of which mainly run a concentric course around the abscess and are continuous with the healthy or slightly inflamed periodontinal membrane higher up the root; this forms the abscess sac, within which the pus is formed. With the first deposition of plastic lymph at the extremity of root, the surrounding bone becomes absorbed to make room for the expanding sac, but the rapidity and extent of this destructive action is very much more accelerated when suppuration ensues, in many cases resulting in the disappearance of a considerable portion of the external or internal plate of alveolus, and the pointing and discharge of the abscess through the gum, or if the periodontinal membrane has been destroyed, the pus finds its way along the surface of the root to the margin of the gum

The intensity of the pain from any given abscess is proportionate, not to the size, but to the intensity of the inflammatory process, the length of the roots, and the resistance to absorption made by the hard tissues.

Symptoms of Alveolar Abscess.—I do not think it necessary to detail these, as we are all quite familar with them, and it would be just a waste of time.

Abscesses originating at the extremities of the incisor and canine teeth of the upper jaw generally perforate the external alveolar wall, the fistulous openings being a little below the focus of suppuration. In some cases the abscess opens into the nasal cavity or on the palate, this last more particularly in connection with diseased laterals.

Abscesses in the incisor teeth of the lower jaw are of less frequency than those of the upper, and in nearly every case the sinus opens on the labial surface Alveolar abscesses connected with the upper premolars and molars usually open upon the facial wall of the jaw, and it is not uncommon to find the roots of several teeth projecting into the cavity of the same abscess. Sometimes the pus, after penetrating the periosteum of jaw, dissects its way along the connective tissue sheaths of the muscles, and escapes through the integument causing buccal fistula, or penetrates the parotid gland, producing salivary fistula. If the extremities of the roots project far into the central cavity, the pus may perforate the mucous membrane and escape into the cavity, or, if the roots are extracted, may give rise to a fistula of the antrum, which is displayed in the specimen I hand round, as a funnel-shaped depression, with a corresponding opening on the floor of the cavity. I have come across and treated several cases of the so-called antral abscess produced by abscessed roots or teeth, and it may interest you to know which produced this lesion in my cases. Four were caused by the first molar, three by the second premolar, one by the first premolar, and one by the lateral incisor ; this last case is interesting from its rarity, and the fact that both anthra became affected, there being an interval of three years between each, the tooth on this other side being the second premolar. With abscesses connected with the molars and premolars of lower jaw there is always great danger of the formation of fistulous tracks along the faciæ, and whether the fistulæ open on the cheek, margin of inferior maxilla, before or behind the ear, upon the nape of the neck or thorax, their true character and significance are too frequently unrecognized, and improper treatment adopted by physicians into whose hands they fall, resulting in permanent deformity to the sufferer, or in some cases even death.

Abscessed third molars, from their position and anatomical relations, are more liable than any other tooth to lead to such results, and this not always from caries and the subsequent destruction of the pulp, as impacted wisdom teeth may also give rise to abscess. I had a good case illustrative of this some months ago,—an old gentleman over 60 years of age was sent to me by his medical adviser in regard to a swelling over the angle of the lower jaw (which had puzzled them very much to account for). I felt sure it was an alveolar abscess, and one that would soon point on the face; the wisdom tooth and second molar were amissing, all the other teeth being sound. However, over the situation where the wisdom tooth should have been there was a small opening in the gum, and on passing a probe into it it came in contact with what I thought was a tooth. Giving a mixture of gas and ether I managed with difficulty to extract the tooth, which was lying parallel to the body of the jaw, it had a large abscess sac attached to the roots, and proved to be the third molar, which had never erupted, the pulp having by some means been destroyed, irritation had been set up, ending in alveolar abscess.

ACUTE ALVEOLAR ABSCESS.—In acute alveolar abscess the disease runs a rapid course, the symptoms for the time being are very severe, but as soon as a fistulous opening is established the pain ceases, and a chronic condition ensues. This, however, only if the attack has been very severe, in milder cases resolution cuts short the attack for the time being; to recur again, the first exciting cause. The abscess sac in this variety is usually thinner, smaller, and smoother than that of the chronic form.

CHRONIC ALVEOLAR ABSCESS.—In chronic alveolar abscess the symptoms are very much less severe; in fact, there may be little or no inconvenience felt, this being very apt to cause the tooth and its diseased conditions to be overlooked and neglected. It is in such cases, in subjects of a strumous diathises, we are liable to have considerable destruction of bone from necrosis or caries. The continual irritation of the periodontinal membrane in chronic alveolar abscess has a peculiar effect on the roots of teeth, causing in some cases the production of layers, of new cemental tissue (destructive,) or removing part of the root by absorption (destructive,) the one frequently alternating with the other at each exacerbation of the disease. In some cases of chronic alveolar abscess there is deposited at the extreme points of the roots nodules of a very hard greenish tartar, which must be derived from the vessels of the abscess sac, and is a condition, so far as I am aware, that is not alluded to in any of the text-books. I hand round for your inspection specimens of this pathological lesion.

Treatment of Alveolar Abscess.—Alveolar abscess has become far more amenable to treatment within the last ten years or so, partly owing to its pathology being better understood, and partly to the discovery of so many useful anti-septics, and the anti-septic system of treatment. Teeth which at one time were condemned to extraction can now, by antiseptic treatment, be saved and made useful again. The anti-septic treatment, however, to do good must be thorough; the saliva and all moisture should be excluded by means of the rubber dam, or other appliances, and this not only at the time when the abscess sac is injected, but at every dressing of the tooth the same precautions should be adopted, otherwise very little good will result.

The treatment to adopt will depend on the stage at which the abscess is seen, and whether it be acute or chronic. To simplify matters it will be as well to divide them into two classes: 1st. Abscess with a pus discharging fistula. 2nd. Abscess sometimes called blind, or those without any external opening. Those of the first class are by far the easiest managed, owing to the readiness with which medicinal agents can be applied to the root of the disease. If the tooth has a filling in it, this should at once be removed, the canals freely opened up and cleaned thoroughly with a weak solution of carbolic acid (a weak solution being used to prevent coagulation of pus cells, if such should be present in the canals,) a drop or two of the same solution should now be inserted into the cavity, and a piece of unvulcanized rubber worked up and down like a piston in the cavity, which generally forces the fluid through the sinus; this having been accomplished, pure carbolic acid (the cheek being protected) may now be used, after which the tooth can be dressed, each root separately with pieces of cotton wool or silk, dipped in what I consider the best and least irritating of anti-septics—a paste of eucalyptus oil and iodoform freshly mixed, and a piece of cotton wool dipped in sandarach varnish applied over this. One or two such dressings usually suffice, although I have filled teeth right away after injecting the sac, and they have done wonderfully well; still it is better practice to give them several dressings before filling, and if need be, another injection of the pure carbolic. Unless the pure carbolic acid passes freely through the sinus, all the dressing that can be applied will do little permanent good, except in a few rare cases. For premolars and single rooted teeth the hypodermic or abscess syringe answers admirably for injecting abscess sacs. Inserting the syringe into the canal of tooth and surrounding the tube with cotton wool dipped in sandarach solution or making it tight any other way, the anti septic fluid can then be readily forced into and through the sinus. In the case of posterior approximal cavities in the molar teeth of upper or lower jaw, I am in the habit of filling up the entrance of the canals with cotton wool, stopping the cavity with amalgam, and when hard, drilling a good sized hole in the centre of the crown, through which the cotton wool can be removed and the tooth very much more readily treated, from the direct manner in which the canals can be reached.

I now come to the second class, viz.:—Abscesses without any fistulous opening. If it be difficult to treat abscesses already fistulous, how much more is it the case with this variety; in fact, these are the most difficult and troublesome cases we have to deal with in practice, and very frequently end in our complete failure to arrest the disease.

There are several methods by which such teeth may be treated. Firstly—By anti-septic dressings applied every two or three days, the administration of a brisk purgative and Sulphate of Quinine in three or four grain doses thrice daily, or Calcium Sulphide in one-tenth grain doses every two hours, either of which have a good effect in promoting the absorption of the inflammatory exudation. Secondly

—Filling the tooth with cement or gutta-percha, and by this means try to produce a fistulous opening. Thirdly—Drilling a hole through the alveolus as near the position of the abscess sac as possible, by means of a drill or small trephine, such as I hand round ; keeping the opening patulous by means of cotton wool tents, or what I find better, a piece of doubled up silver suture wire, which should be allowed to remain till the canals of tooth are thoroughly disinfected, and the pus and exudate drained away. Each and all of these methods I employ according to the exigencies of the case, but one has to confess that our methods of treatment are not so accurate, but that we have numerous failures in this class of cases ; the principal cause of such being our inability to get a free opening through the canals of the tooth into the abscess sac and sinus, artificial or otherwise.

In cases of alveolar abscess which threaten to open on the face, the tooth should at once be extracted, and if there has been any infiltration of the surrounding tissues, it is a good plan to adopt the precaution of cutting across the fistulous canal, otherwise, even although the tooth is extracted, the infiltrated pus may find its way to the face, and produce a sinus. After extracting abscessed premolar or molar teeth in the lower jaw we sometimes find the swelling increasing afterwards to a great size, and bulging out the external alveolar plate, this being caused by the infiltration into the cancellated structure of the bone, and the rapid proliferation of the pus cells. I have found it useful in such cases to pass a trochar into the socket and open it up freely, so as to drain away the inflammatory exudate. The old and very unscientific operation of rhizodontresis, was performed to prevent, or rather hide, alveolar abscess in teeth, the pulps of which had been destroyed by arsenious acid, and usually not removed. This may be all very well as a temporary expedient in stopped teeth, the pulps of which have subsequently become dead and threaten alveolar abscess ; but to do so in every case cannot be too strongly condemned, and I pity the poor patient who has a number of such teeth, draining the contents of their abscesses through the artificial opening in the tooth into his stomach.

A very prominent question at the present time among pathologists, and one which is of great interest to us dental surgeons, is—"Do the presence of micro organisms spread and produce or intensify inflammatory action?" That they are the origin of inflammation very few believe, but when they are present they are apt to infect the surrounding structures, and thus spread and intensify the inflammation. In the case of alveolar abscess, with a fistula and a carious open cavity, we can readily understand microzymes to be present in abundance, but one is inclined to doubt their presence in abscesses connected with perfectly sound teeth, the pulps of which have been destroyed by a blow or fall, or in a tooth which has been filled for a number of years, the pulp having been unexposed, but which subsequently has become dead and produced abscess. I recently examined the pulp of one such tooth which had caused abscess, and found it white and sodden, but without any putrid odor, and, so far as I could make out, there were no micro-organisms present. This subject, however, requires patient and thorough investigation before we can tell definitely the effect which micro-organisms have in inflammatory lesions. I have prepared, and will show, several slides with pus taken from abscesses, acute and chronic, the first by asperations from an unopened abscess, the second from a fistulous abscess, and being stained by the most approved method, they, in both cases, show the presence of micrococci and bacteria in considerable numbers.

At the conclusion of Mr. Watson's paper and demonstrations, the Chairman, who had to leave the meeting earlier this evening, said that he hoped a full discussion would take place on the very interesting communication which had just been laid before the Society. Three points in it presented themselves for consideration. These were—first, the great number and peculiarities of the structures implicated in alveolar abscess; second, the special characters and distinctive nature of those morbid affections occurring among them, as compared with inflammatory action and its results in other similar tissues, such as those found associated with joints, etc.; and third, the important part played by such agencies as Micrococci, Bacteria, Bacilli, those interesting novelties of late occupying the attention of pathologists and other scientific inquirers. With reference to anti-septic treatment in the strict sense of the phrase, applied in oral surgery of any kind, this could scarcely be carried out in the Listerian point of view—since the adoption of all its essential requirements is here all but impossible. But of the therapeutic, if not the physiological value, of the action of such anti-septic agents as has been mentioned by Mr. Watson, there could not be a doubt.

Dr. Smith then left the chair, which was taken by Mr. Wilson.

Mr. Finlayson thought that no exception could be taken to the scientific part of the paper, which, with the microscopic specimens submitted to inspection, had been evidently got up with care, and reflected great credit on the author. He (Mr. Finlayson) had not seen a case of metastasis such as was referred to, viz., pointing of alveolar abscess in the inner portion of the lower eyelid. With regard to the treatment in cases, he might mention that one he had brought under the notice of the Society in December, and which they would recollect had been treated by being washed carefully out every day with weak carbolic solution, and thereafter the distended walls of the alveolus packed with boracic lint, the first packing being on November 27th, and the capacity of the distended cavity being equal to two fluid drachms. This was done regularly every day, the quantity of packing being gradually decreased, until, at this date, only one fourth the bulk is needed to fill it up. Several sequestra of bone, very thin, had come away, but those he had not seen. The pus had a very light smell, and had along been as nearly as possible laudable. Boracic lint, as an anti-septic dressing, had in this case proved very useful and non-irritating, as granulation appeared to have gone on steadily from the first.

Mr. AMOORE said, Mr. Watson had referred to a subject which was powerfully exercising the minds of pathologists of the present day—the influence of micro-organisms in the production of disease. He had lately been reading the lectures on inflammation by Prof. Burdon Sanderson referred to, and quoted by Mr. C. Tomes in a recent paper read before the British Dental Association at Liverpool. Mr. Watson had stated it to-night as his opinion that some abscesses, namely, those that had no communication with the free surface, were not influenced in their growth by microzymes. This somewhat accorded with Mr. Tomes' views, for in the few cases that he had investigated where the pulps of teeth had died, and in some instances undergone putrefactive decomposition without communication with the exterior, he had not as yet satisfactorily proved the presence of bacteria, or of micrococci in the contents of the pulp-chamber. Professor Burdon Sanderson had entered at some length into experiments performed upon rabbits, which went to show that inflammation-producing organisms "do not exist in the atmosphere, nor in the ordinary aqueous liquids with which our bodies come in contact," and that these micro-organisms are not primary causes of inflammation, but are rather "mischief spreaders" than "mischief makers." An illustration which Mr. Tomes had quoted was a very instructive one, and though it was doubtless known to most of those present, he might perhaps be permitted to briefly repeat it. The method of castrating male animals in the South of France differed from that commonly pursued here. The testis is twisted upon the spermatic cord, so as to completely cut of all vascular connection without injury to the overlying skin. The results are almost invariably favorable, and the dislocated organ undergoes atrophy, the result of this simple inflammation being confined to the directly injured tissue. But perhaps the most instructive part is to follow. Several animals, previous to the operation, were injected with septic matter —pus from an infective abscess—and in the majority of cases death resulted from septicœmia. Mr. Tomes considered the relative condition of a pulp, severed from its external vascular connections by a blow upon the surface of the tooth, as precisely analogous to that of the dislocated testis in the foregoing operation. A tooth that has thus suffered injury from a blow may remain useful for a long period, or, as frequently happens, after an interval of variable duration, the periosteum becomes inflamed, and abscess ensues. Now, if this is a result arising from decomposition of the pulp, and infection of the tissue therefrom, the analogy is not born out, and it is difficult to account for the abscess; but, if there be infective material in the blood during the period of inflammation of the periosteum, it appeared to him to be more readily comprehensible, as the microzymes would there find suitable "soil" wherein to grow and flourish. He feared he had only repeated what must have been their own conclusions on reading the papers referred to, but he trusted, as the matter had not been brought forward that night, that its important connections with the subject in hand were sufficient apology.

Mr. Macleod said he had listened with some interest and attention to Mr. Watson's paper, and while admitting that in many respects it was a most creditable one, he was constrained to express the disappointment which he felt at its conclusion, in so far that there was nothing in the paper which had not been already most fully discussed at the meeting set apart for that purpose in January, 1881. Now, he was rather of opinion that some advance had been made in our knowledge of the pathology of alveolar abscess, and certainly there had been some contributions to our more exact treatment of this disease, and to these he had expected Mr. Watson would have turned their attention. He (Mr. Watson) certainly made allusion to micro organisms, but did not venture to express an opinion as to the likelihood of their presence being antecedent, concurrent, or subsequent to the inflammatory stage, or if only present when the diseased structure was or had been in contact with the air. Now, the specimen shown by Mr. Watson of abscess in connection with the unerupted wisdom tooth should have formed an excellent text upon which a few thoughts regarding the evolution of micrococci, etc., might have been hung. Mr. Watson in his paper had made reference to nodules of green tartar at the apices of roots, which were the seat of chronic abscess, and said that its presence was rare, and had never been noticed in text-books. As he (Mr. Macleod) did not know to what books Mr. Watson limited the term, of course he could not say that this was not so, but he could assure Mr. Watson that the presence of green tartar at the ends of the roots, under the circumstances mentioned, was by no means so rare as he seemed to think, and that he would find on more extended reading that its presence was mentioned by more than one author, and still more frequently in the journal literature by writers on the so-called Rigg's Disease. That it is a deposit from the "vessels of the abscess sacs" he very much doubted, preferring to concur with older observers and writers on the subject, that its presence is accounted for by the saliva oozing through the canal or passing between the now disconnected peri-cementum and the tooth. In Mr. Watson's paper there was only one original point, and that was in the treatment of alveolar abscess, and to that he would refer, but unfortunately to take exception to it. He alluded to the filling up the pulp cavity with cotton wool, building up the tooth with amalgam, and, when hard, drilling a hole through the amalgam, and picking out the wool, and then treating through the hole thus drilled. He might suggest to Mr. Watson to try two or three strands of well waxed silk, packing the canals and cavity with these, and leaving the ends sufficiently long to extend below the surface of the crown. When the amalgam set the free ends could be seized and the silk pulled out. This would be found more reliable than the cotton wool business; at the same time he was aware that there were simpler ways, and preferred them. In concluding, Mr. Macleod made some remarks on a form of disease which had occasionally come under his notice, and asked if it might be a low form of abscess. The presence of the disease could, as far as knew, only be ascertained by diagnostic exclusion. Its symptoms were neuralgia, and the seat of pain varied with the tooth or teeth attacked, but was seldom present in the tooth itself, and if ever so, only as a feeling of tension passing between the tooth and the seat of pain. On extraction, the pulp was in an atrophied condition, pale in color, and the chamber and canal filled with a thin fluid. The root, to generally about one-third or one-half of its length, was quite translucent, this transluency arising very probably from this portion of the root being bathed in the same fluid found in canal, and being deprived of its organic constitutents.

Mr. WILSON said he agreed very much with the remarks already made. As to the so-called green tartar at the apex of a root, he certainly had seen it noticed, and if he recollected it rightly, a well-marked case had been shown at a meeting of this Society some years ago, the production being supposed to be from an infiltration of saliva into the enlarged socket, a cause which seemed to him rather unlikely. As regarded micro-organisms in the oral cavity, and their action on the teeth, whether these were healthy or carious, he recommended those members who had not already done so, to read the able article on that subject in the *Cosmos* for January, by M. Miller D. D. S., Berlin.

Mr. WATSON in reply, said—Mr. Macleod in his remarks mentions that I have not gone so deeply into the subject of micro-organisms, and the effect they produce in inflammatory conditions, such as that of alveolar abscess. I have not done so, for the simple reason that I do not wish to dogmatise on a subject I know very little about, and one which only two or three of the most eminent histologists can give any information about. He also says that the presence of alveolar abscess in connection with the impacted tooth referred to in my paper, is a proof that micro-organisms were present; this I utterly deny, as any one who has a knowledge of pathology knows, that abscess can arise independently of the presence of micro-organisms. Mr. Macleod has evidently understood me in the treatment I adopt with difficult posterior approximal cavities in molars. After filling permanently the posterior approximal cavity, I drill a hole in the centre of the crown to get direct access to the canals in the treatment of the abscess. He also seems not quite to take me up in regard to the deposit of nodules of tartar at the extremities of roots in some cases of chronic alveolar abscess, as this deposit differs entirely from that of Rigg's disease, which is found in the shape of scales scattered over the surface of the root, and not localised to the one spot, as in my specimens.

Mr. Macleod claimed a word of explanation. He did not say that the presence of alveolar abscess in connection with the tooth referred to was a proof of the presence of micro-organisms, but that the presence or non-presence of these in this particular abscess would have afforded argument for or against the theory which attributed the origin of this pathological condition to microzymes. Nor did he say that green tartar at the end of the root was an accompaniment of Rigg's disease, but that its existence had been incidentally mentioned by certain authors when writing on that disease.

On the motion of the Chairman, a vote of thanks was accorded to Mr. Watson.

The Chairman then intimated that the next meeting, being the Annual Meeting, would be held on Tuesday, the 13th of March, when several interesting communications would be laid before the Society, and he hoped that every member would make a strong effort to be in his place.—*Dental Record.*

